# Full-length and C-terminal neurogranin in Alzheimer’s disease cerebrospinal fluid analyzed by novel ultrasensitive immunoassays

**DOI:** 10.1186/s13195-020-00748-6

**Published:** 2020-12-22

**Authors:** Annika Öhrfelt, Julien Dumurgier, Henrik Zetterberg, Agathe Vrillon, Nicholas J. Ashton, Hlin Kvartsberg, Elodie Bouaziz-Amar, Jacques Hugon, Claire Paquet, Kaj Blennow

**Affiliations:** 1 Department of Psychiatry and Neurochemistry, Institute of Neuroscience and Physiology, The Sahlgrenska Academy at the University of Gothenburg, Sahlgrenska University Hospital/Mölndal, SE-431 80 Mölndal, Sweden; 2 Université de Paris, INSERM U1144, Center of Cognitive Neurology, Lariboisière – Fernand-Widal Hospital, APHP, Paris, France; 3 Clinical Neurochemistry Laboratory, Sahlgrenska University Hospital, Mölndal, Sweden; 4 Department of Neurodegenerative Disease, UCL Institute of Neurology, London, UK; 5 UK Dementia Research Institute, London, UK; 6 Wallenberg Centre for Molecular and Translational Medicine, University of Gothenburg, Gothenburg, Sweden; 7 Department of Old Age Psychiatry, Institute of Psychiatry, Psychology and Neuroscience, King’s College London, London, UK; 8 NIHR Biomedical Research Centre for Mental Health and Biomedical Research Unit for Dementia at South London and Maudsley NHS Foundation, London, UK; 9 Department of Biochemistry, Lariboisière – Fernand-Widal Hospital, Université de Paris, INSERMU1144, APHP, Paris, France

**Keywords:** Alzheimer’s disease, Biomarkers, Cerebrospinal fluid, Mild cognitive impairment, Neurogranin, Single molecule array, SNAP-25, Synaptic, Synaptotagmin-1

## Abstract

**Background:**

Neurogranin (Ng) is a neuron-specific and postsynaptic protein that is abundantly expressed in the brain, particularly in the dendritic spine of the hippocampus and cerebral cortex. The enzymatic cleavage of Ng produces fragments that are released into cerebrospinal (CSF), which have been shown to be elevated in Alzheimer’s disease (AD) patients and predict cognitive decline. Thus, quantification of distinctive cleavage products of Ng could elucidate different features of the disease.

**Methods:**

In this study, we developed novel ultrasensitive single molecule array (Simoa) assays for measurement of full-length neurogranin (FL-Ng) and C-terminal neurogranin (CT-Ng) fragments in CSF. The Ng Simoa assays were evaluated in CSF samples from AD patients (*N* = 23), mild cognitive impairment due to AD (MCI-AD) (*N* = 18), and from neurological controls (*N* = 26).

**Results:**

The intra-assay repeatability and inter-assay precision of the novel methods had coefficients of variation below 7% and 14%, respectively. CSF FL-Ng and CSF CT-Ng median concentrations were increased in AD patients (6.02 ng/L, *P* < 0.00001 and 452 ng/L, *P* = 0.00001, respectively) and in patients with MCI-AD (5.69 ng/L, *P* < 0.00001 and 566 ng/L, *P* < 0.00001) compared to neurological controls (0.644 ng/L and 145 ng/L). The median CSF ratio of CT-Ng/FL-Ng were decreased in AD patients (ratio = 101, *P* = 0.008) and in patients with MCI-AD (ratio = 115, *P* = 0.016) compared to neurological controls (ratio = 180). CSF of FL-Ng, CT-Ng, and ratio of CT-Ng/FL-Ng could each significantly differentiate AD patients from controls (FL-Ng, AUC = 0.907; CT-Ng, AUC = 0.913; CT-Ng/FL-Ng, AUC = 0.775) and patients with MCI-AD from controls (FL-Ng, AUC = 0.937; CT-Ng, AUC = 0.963; CT-Ng/FL-Ng, AUC = 0.785).

**Conclusions:**

Assessments of the FL-Ng and CT-Ng levels in CSF with the novel sensitive immunoassays provide a high separation of AD from controls, even in early phase of the disease. The novel Ng assays are robust and highly sensitive and may be valuable tools to study synaptic alteration in AD, as well as to monitor the effect on synaptic integrity of novel drug candidates in clinical trials.

## Background

Alzheimer’s disease (AD) is the most common cause of dementia. The disease is characterized by the accumulation of extracellular amyloid-β (Aβ) plaques, intra-neuronal neurofibrillary tangles (NFT), along with substantial neuronal degeneration and synaptic loss [1]. Numerous studies have consistently shown that the core AD cerebrospinal fluid (CSF) biomarkers (Aβ_1–42_, T-tau, and P-tau) have a high diagnostic accuracy in AD dementia and also in preclinical stages of disease [2, 3]. In recent years, many synaptic proteins, including neurogranin (Ng), synaptosomal-associated protein 25 (SNAP-25), and synaptotagmin-1, have been suggested to be biomarkers reflecting synaptic degeneration and synaptic loss. The CSF levels of these synaptic proteins are markedly elevated in AD dementia, and mild cognitive impairment with dementia due to AD (MCI-AD) [4–8]. Furthermore, Portelius et al. have shown a link between CSF Ng levels and neuropathological lesions including neuritic plaques and levels of NFT with Braak stages highlighting its link to the pathophysiology of AD [9]. Synaptic pathology occurs early in the AD brain [10, 11] and have shown to be reduced in hippocampus and cortical regions, while unchanged in cerebellum, a non-affected AD region [12–14]. The synaptic functioning is the support of cognition and therefore synaptic degeneration and synaptic loss might be much closer related to cognitive decline than the other pathological hallmarks of AD [10, 11, 15].

Ng is a 78-amino-acid (aa)-long postsynaptic protein which is highly expressed in dendritic spines in the central nervous system (CNS) [16, 17] and plays a pivotal role in the synaptic plasticity [18, 19]. Synaptic activity, mediated by Ca^2+^ influx, regulates synaptic plasticity through the calcium-binding protein calmodulin [19], which at high concentrations of Ca^2+^ activates several enzymes that are essential for long-term potentiation (LTP) [20, 21]. At low intra-neuronal levels of Ca^2+^, calmodulin associates to the IQ motif (aa 33–43) (IQASFRGHMAR) of Ng [22, 23] that are thought to lower the threshold for the induction of LTP [20]. In contrast, postsynaptic activation raises the local concentration of Ca^2+^, leading to phosphorylation of a serine residue (Ser36) within the IQ motif, rendering the binding to calmodulin impossible [24, 25]. Thus, LTP is initiated by transiently Ca^2+^ influx inducing Ng dissociation from calmodulin and is essential for establishment of memory and cognition in hippocampal neurons [17, 20, 26]. In the pre-synapse, synaptotagmin-1 and SNAP-25 are two key players for properly synaptic vesicle exocytosis mediated neurotransmitter release and synaptic activation [27–30]. Postsynaptic exocytosis machinery failure may consequently account for neuronal postsynaptic disturbances, and vice versa, that might cause synaptic malfunctioning experienced in AD [29, 31].

We have previously shown by combining immuno-enrichment and mass spectrometry that Ng is cleaved into a series of C-terminal peptides before release into the CSF [4, 32]. Most of the identified peptides are in close proximity of the C-terminal end of the IQ motif of Ng, and terminate at aa 75, 76, or 78 [32]. Recently, we identified two enzymes, Calpain-1 and prolyl endopeptidase, that are capable to generate some of the Ng C-terminal peptides found in CSF [32, 33]. So far, our in-house designed quantitative immunoassays have only included antibodies against the C-terminal half of Ng (CT-Ng) lacking IQ motif [4, 9, 32, 34]. All these studies have consistently shown increased levels of CT-Ng in AD [4, 9, 32, 34]. Other assays are based on antibody combinations targeting full-length Ng (FL-Ng), showing similar increases in AD [35, 36].

Elevated levels of synaptic proteins in CSF are thought to reflect the loss of synapses in AD [37, 38]. Most of the synaptic AD biomarkers investigated up to now, i.e., Ng, synaptotamin-1, and SNAP-25, are present in CSF as apparently shorter fragments [5, 6, 32]. Targeting of distinctive parts of CSF proteins and post-translational modifications can shed light upon pathological aspects of disease. As an example, we showed that CSF tau fragments correlated with tau positron emission tomography (PET), suggesting that these fragments might reflect the tangle pathology [39]. Another example is the CSF ratio of Aβ_1–42_/Aβ_1–40_ that have shown to perform diagnostically better than the levels of CSF Aβ_1–42_ alone [40]. Therefore, it would be of interest to investigate if normalization of the CT-Ng concentrations to FL-Ng would improve the diagnostic accuracy. Despite of the fact that the C-terminal Ng species seems to be the vast majority of Ng species, we have shown by combining immuno-enrichment and Western blotting that Ng is present as a seemingly FL-Ng protein in CSF [41]. However, so far, no quantitative in-house immunoassay for assessment of human FL-Ng that includes the IQ motif in CSF samples has been available. We anticipated considerably low levels of FL-Ng in CSF, thus we employed the single molecule array (Simoa) technology to provide better sensitivity than a conventional enzyme-linked immunosorbent assay (ELISA) [42].

The aim of this study was to develop and investigate CSF FL-Ng and CT-Ng to evaluate if these Ng species, alone or in combination, can reveal features of AD pathology. Novel Simoa immunoassays quantified FL-Ng and CT-Ng in parallel in human CSF samples. We tested the hypothesis that CSF Ng concentrations and the ratio of CT-Ng/FL-Ng would significantly differ along the AD continuum. Furthermore, we evaluated if the CT-Ng/FL-Ng ratio would yield an improved diagnostic accuracy for AD as compared to single measures of CT-Ng and FL-Ng. We also evaluated if the CSF Ng levels have associations with the core CSF biomarkers, presynaptic CSF biomarkers (SNAP-25 and synaptotagmin-1) and cognition, as measured by the Mini-Mental State Examination (MMSE).

## Methods

### CSF samples in the clinical study

In this cross-sectional study design, FL-Ng and CT-Ng levels in CSF were measured in a clinical patient cohort consisting of patients with AD (*N* = 23), MCI-AD (*N* = 18), and neurological controls (*N* = 26). The clinical and demographic characteristics and the biomarker CSF levels of the AD core biomarkers for the cohort have been partially reported previously [6] and further detailed below. At the Center of Cognitive at Lariboisière Fernand-Widal University Hospital APHP, patients underwent a thorough clinical examination involving personal medical and family histories, neurological examination, neuropsychological assessment, and lumbar puncture with CSF biomarker analysis. The diagnosis for each patient was made by neurologists considering CSF results and according to validated clinical diagnostic criteria for AD [43], MCI-AD [44, 45], subjective cognitive impairment [46], and psychiatric disorder (DSM-IV). The CSF samples of the study were selected after a second validation step by a neurologist (CP) and a biochemist (EAB). Patients were not included in the study if a consensus diagnosis has not been achieved. This procedure resulted in the selection of CSF samples from subjects with MCI-AD, AD, and neurological controls (no neurodegenerative disorders).

### Analysis of CSF core biomarkers

CSF was obtained by lumbar puncture between the L3/L4 or L4/L5 intervertebral space, and samples were immediately centrifuged at 1800*g* for 10 min at + 4 °C and stored at − 80 °C pending analysis. The Aβ_1–42_, T-tau, and tau phosphorylated at threonine 181 (P-tau) protein measurements were previously performed using commercially available assays from Fujirebio (INNOTEST® β-AMYLOID_(1–42)_, INNOTEST® hTAU Ag, and INNOTEST® PHOSPHO-TAU(181P) according to the manufacturer’s instructions [6]. The following cut-off values were used to define a biochemical AD signature as supportive criteria for AD [43]: Aβ_1–42_ (< 730 ng/L), T-tau (> 340 ng/L), and P-tau (> 58 ng/L).

### Analysis of SNAP-25 and synaptotagmin-1

The CSF levels of the pre-synaptic markers SNAP-25, synaptotagmin-1 peptide 215–223 (VPYSELGGK), and synaptotagmin-1 peptide 238–245 (HDIIGEFK) have previously been analyzed with an ELISA and a mass spectrometry-based assay [6, 7]. In this study, we investigate the relation between the CSF levels of FL-Ng and CT-Ng and also the relation to the pre-synaptic markers.

### Production of anti-Ng antibodies and recombinant Ng protein

The monoclonal NGN1, NGN2, NGN3, and NGN4 antibodies were produced by immunization of 8-week-old *BALB/c* mice with KLH-conjugated peptides (Caslo, Lyngby, Denmark) in Freund’s adjuvant, Complete (Sigma-Aldrich, F5881). The peptide sequence of the immunogen was Ac-(KLH)-CSKPDDDILDIPLDDPG-NH, i.e., aa 9–25 of Ng. After 2–3 dosages with the immunogen (approximately 100 μg/mice) in Freund’s adjuvant, Incomplete (second dose), or in phosphate-buffered saline (PBS) (third dose), the spleen was removed and B cells were fused with the myeloma cell line SP2/0 accordingly to standard procedures. Approximately 10 days after fusion of cells, cell media were screened for Ng antibodies using the unconjugated Ng immunogen peptide. Clones that reacted with the immunogen peptide, but not with a negative control peptide, were further grown, sub-cloned, and subsequently frozen in liquid nitrogen. The antibody specificity was tested using unconjugated Ng peptides (aa 9–25, aa 1–20, aa 15–34, and recombinant full-length Ng protein). The isotype was determined using the Pierce™ Rapid Isotyping Kit-Mouse, accordingly to the manufacturer’s instructions. Then, the antibodies were purified using HiTrap™ Protein G HP columns (GE Healthcare).

The generation of monoclonal anti-Ng antibodies Ng2 and Ng36 has been described previously [4, 47]. The Ng2 antibody recognizes an epitope within the aa 52–63 of Ng [4], while the Ng36 was generated with KLH-conjugated peptide Ng 63–75 as immunogen. The production of GST tag full-length Ng calibrator has been described previously [41].

### Novel Simoa assays for CSF FL-Ng and CT-Ng

The Ng Simoa methods were established using guidelines found in the Homebrew Assay Development Guide Simoa HD-1 Analyzer & Quanterix SR-X guide (Quanterix, Boston, MA, USA). Briefly, carboxylated capture beads (Quanterix) were activated for 30 min at + 2–8 °C by adding 0.3 g/L (FL-Ng) and 0.2 g/L (CT-Ng) Pierce™ 1-ethyl-3-[3-dimethylaminopropyl] carbodiimide hydrochloride (EDC), No-Weigh™ Format (Thermo Scientific, A35391) to beads (Quanterix, 103207) solution of 1.4 × 10^6^ beads/μL. Then, the beads were washed using a magnetic separator. The monoclonal capture antibodies (0.2 g/L of NGN4, FL-Ng assay, and 0.2 g/L of Ng36, CT-Ng assay) were allowed to conjugate to the activated beads for 2 h at + 2–8 °C. The conjugated capture beads were blocked and stored at + 2–8 °C pending analyses. The detection antibody was prepared by biotinylating the monoclonal antibody Ng2 in 40-fold-molar-excess of EZ-Link NHS-PEG4-Biotin (Thermo Scientific, 21329). Bead reagent solution was prepared by mixing helper beads (Quanterix, 103208) and antibody conjugated capture beads in a proportion of 50:50 (500 K and 500 K). The beads were washed two times in PBS containing 0.05% Tween20 and 1% bovine serum albumin (BSA) (Sigma, A7906) (0.05%) (PBS-T) (assay diluent) and re-suspended in assay diluent, leading to a total concentration of 20,000 beads/μL. All calibrators and CSF samples were analyzed in duplicate. For each run, human full-length GST-tagged recombinant Ng calibrator, produced in-house [41], was serially diluted in assay diluent to providing a final concentration range of 312.5–2.44 ng/L and 1250–4.88 ng/L for FL-Ng assay (Capture NGN4 and Detection Ng2) and CT-Ng assay (Capture Ng36 and Detection Ng2), respectively. Detector Ng2 reagent solution was prepared by dilution of biotinylated detection antibody to 0.736 μg/mL in assay diluent. Both Ng immuno-methods were run in 2-step assay format. For the FL-Ng assay and CT-Ng assay, 100 μL (neat CSF sample or diluted in 1:5 in assay buffer, respectively) and 100 μL calibrator were added to the Nunc™ 96-Well Polypropylene Storage Microplates (Thermo Scientific, 249944). Samples and calibrators were incubated for 2 h at 800 rpm on a Simoa microplate shaker (Quanterix), simultaneously with 75 μL detector reagent solution and 25 μL bead reagent solution. Then, the post-detector washing program on the Simoa microplate washer (Quanterix) was applied. After washing, 100 μL of streptavidin-conjugated β-galactosidase (SBG) (Quanterix) at 150 pM diluted in SBG diluent (Quanterix) was added and incubated for 10 min at 800 rpm on a Simoa microplate shaker. Then, the Post-SBG + Buffer B Wash 2.0 program on the Simoa microplate washer was applied. The bead pellets were allowed to dry for 10 min prior to analysis on a Quanterix SR-X benchtop instrument. Prior to measurements, the vials of resorufin β-D-galactopyranoside (RBG) (Quanterix) were shaken at room temperature for 2–3 h. The sample concentrations of CSF Ng were calculated from the four-parameter logistic standard curve. Calibration curve data from five runs were used to validate the assays. Limit of detection (LOD) was determined as 3 standard deviations above the zero calibrator. All unknown samples, standard samples, and human quality control (QC) CSF samples were run at least in duplicates.

### Performance of FL-Ng and CT-Ng Simoa assays

The repeatability of the Ng Simoa assays was examined on anonymized human CSF samples supplied by the clinical routine section at the Clinical Neurochemistry Laboratory, The Sahlgrenska University Hospital, Mölndal, Sweden. The procedure making pools of leftover CSF aliquots were approved by the Ethical Committee at University of Gothenburg.

The intra-assay repeatability (within-run precision) and the inter-assay precision (between day repeatability) were validated by repeated measurements of human CSF QC (QC1 and QC2) samples at five different occasions. Analysis of variance (one-way ANOVA) was used in the estimation of the imprecision using the formulas in ISO 5725-2. Precision is reported as the inversely related imprecision measure [48].

### Statistical analysis

Because most of the analytes were not normally distributed (Shapiro-Wilk test, *P* < 0.05), non-parametric statistics were used for analysis. Data are given as median (interquartile range). Differences between more than two groups were assessed with Kruskal-Wallis test. Statistically significant results (*P* < 0.05) were followed by Mann-Whitney *U* tests to investigate group differences. Significance values were adjusted by the Bonferroni correction for multiple tests. Receiver operating characteristic (ROC) curves were performed on each subject group on the levels of Ng in order to assess its diagnostic value. The area under the curve (AUC) and a 95% confidence interval (CI) was calculated for CSF Ng using GraphPad Prism 8.01. The correlation coefficients (rho) were calculated using the Spearman two-tailed correlation test. SPSS 24 was employed for most of the statistical analyzes.

Since the controls were significantly older than the MCI-AD group, linear mixed models included CSF Ng (FL-Ng and CT-Ng, respectively) as the dependent variable and the diagnostic groups as a fixed variable, and age as a covariate was preliminary applied. The likelihood ratio test was used to compare the linear mixed models using SPSS 24 and the Chi-Square Calculator (https://www.fourmilab.ch/rpkp/experiments/analysis/chiCalc.html). Because age did not affect the results of any of the linear mixed models (data not shown), the group differences were calculated as described above.

## Results

### Characterization of N-terminal Ng antibodies

All of the in-house produced N-terminal Ng antibodies (NGN1, NGN2, NGN3, and NGN4) were determined to be of mouse IgG_1_ isotype.

### Performance of FL-Ng and CT-Ng Simoa assays

The novel Simoa assays are directed against epitopes within the C-terminal of Ng (CT-Ng, approximately aa 52–75) and against the N-terminal to C-terminal of Ng (FL-Ng, approximately aa 9–63). For the FL-Ng assay, intra-assay repeatability was 6% for QC1 and 4% for QC2, and inter-assay precision was 12% (QC1) and 14% (QC2). For the CT-Ng assay, intra-assay repeatability was 7% (QC1) and 7% (QC2), and inter-assay precision was 12% (QC1) and 11% (QC2). The repeatability were within acceptable ranges, i.e., intra-assay repeatability ≤ 10 and inter-assay precision ≤ 15 [49]. LOD was 0.513 ng/L for the FL-Ng assay and 0.111 ng/L for the CT-Ng assay. Individuals having lower FL-Ng levels than LOD were set to LOD (i.e., 0.513 ng/L). Twelve control participants had FL-Ng levels below the LOD.

### Demographics of the clinical CSF study

A subset of patients from [6] were included in this study; 26 neurological controls (65% women, 43–80 years), 18 patients with MCI-AD (72% women, 58–83 years), and 23 AD patients (74% women, 52–84 years) (Table 1). The AD patients had significantly lower MMSE levels compared to the controls (*P* < 0.0001) and patients with MCI-AD (*P* = 0.00002), respectively. Patients with MCI-AD were significantly older than the controls (*P* = 0.007).

**Table 1:** Demographic data and core AD biomarker levels for the clinical study^a^

	Control	MCI-AD	AD
Number (Women/Men)	26 (17/9)	18 (13/5)	23 (17/6)
Age (years)	62 (53–69), *P* = 0.007^c^	70 (69–78), *P* = 0.007^b^	68 (64–72)
MMSE	27 (25–28)	27 (26–28)	22 (16–24), *P* < 0.00001^b^, *P* = 0.00002^c^
Aβ_1–42_ (ng/L)	970 (811–1091), *P* = 0.00002^c^	558 (377–667), *P* < 0.00002^b^	496 (459–568), *P* < 0.00001^b^
T-tau (ng/L)	182 (154–207), *P* < 0.00001^c^	570 (520–717), *P* < 0.00001^b^	618 (527–734), *P* < 0.00001^b^
P-tau (ng/L)	35 (30–41), *P* < 0.00001^c^	86 (79–104), *P* < 0.00001^b^	90 (73–108), *P* < 0.00001^b^

^a^Data are given as median (interquartile range) unless otherwise indicated. Statistical differences were determined using non-parametric tests. The demographic data and the core AD biomarkers have partially previously been reported [6]

^b^Compared with controls

^c^Compared with MCI-AD

### CSF Ng in the clinical cohort

**Fig. 1:**
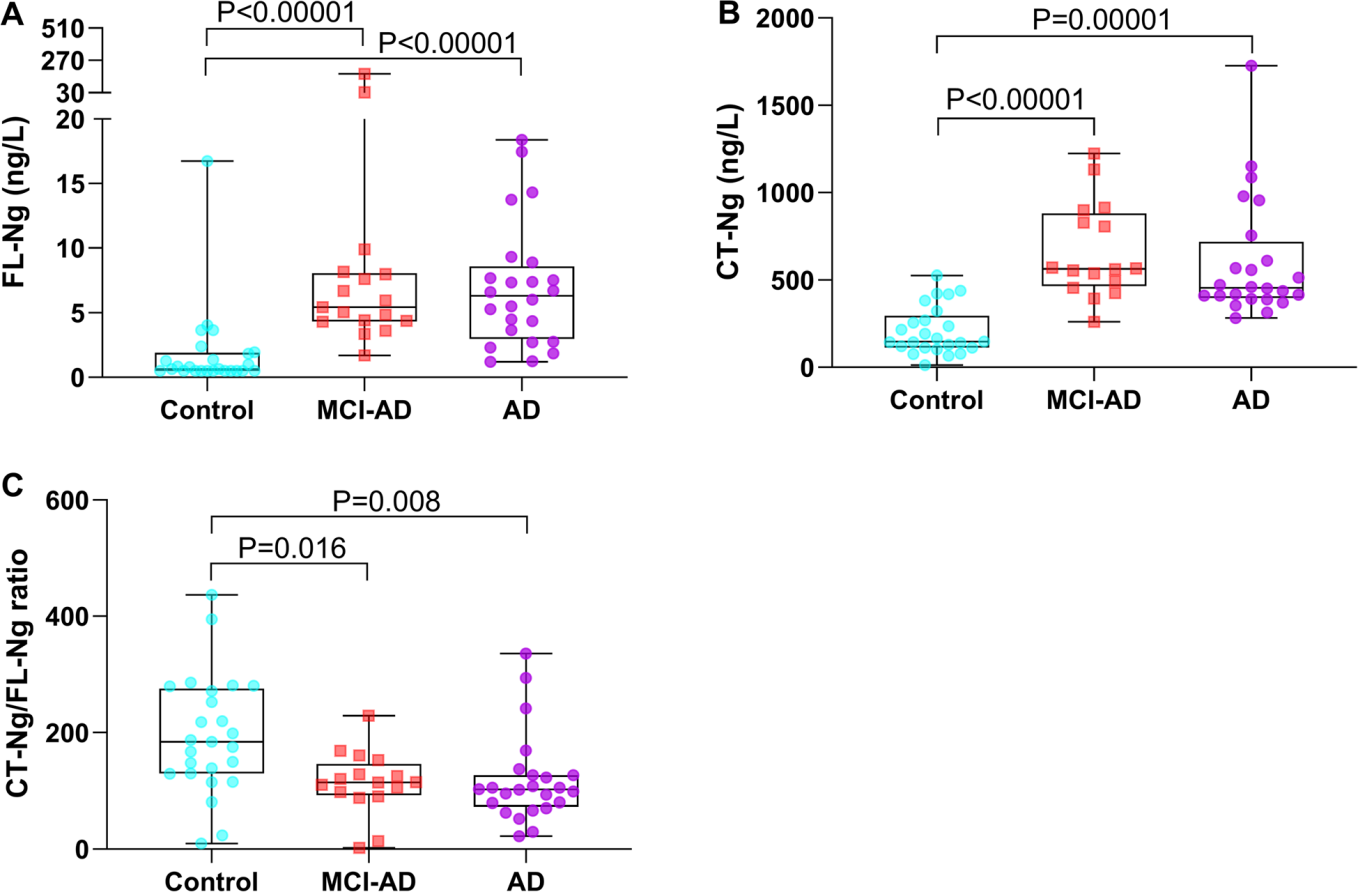
Individual values and box whisker plots for full-length neurogranin (FL-Ng) (**a**), C-terminal half of Ng (CT-Ng) (**b**), and the ratio of CT-Ng/FL-Ng (**c**) in the cerebrospinal fluid (CSF) samples from Alzheimer’s disease (AD) patients (violet), mild cognitive impairment due to Alzheimer’s disease (MCI-AD) patients (orange) and control (turquoise) individuals. The lower, upper, and middle lines of the box correspond to the 25th and 75th percentiles and medians, respectively. The whiskers correspond to the minimal and maximal values

The CSF levels of both FL-Ng and CT-Ng were significantly elevated in patients with MCI-AD (5.69 ng/L, *P* < 0.00001 and 566 ng/L, *P* < 0.00001, respectively) and AD (6.02 ng/L, *P* < 0.00001 and 452 ng/L, *P* = 0.00001, respectively) compared with controls (0.644 ng/L and 145 ng/L, respectively) (Fig. 1). The CSF ratio of CT-Ng/FL-Ng was significantly decreased in patients with MCI-AD (ratio = 115, *P* = 0.016) and in AD (ratio = 101, *P* = 0.008) compared with controls (ratio = 180) (Fig. 1). In the group of controls, MCI-AD, and AD, respectively, the median levels of CT-Ng were 225, 100, and 75 times higher than the median levels of FL-Ng.

**Fig. 2:**
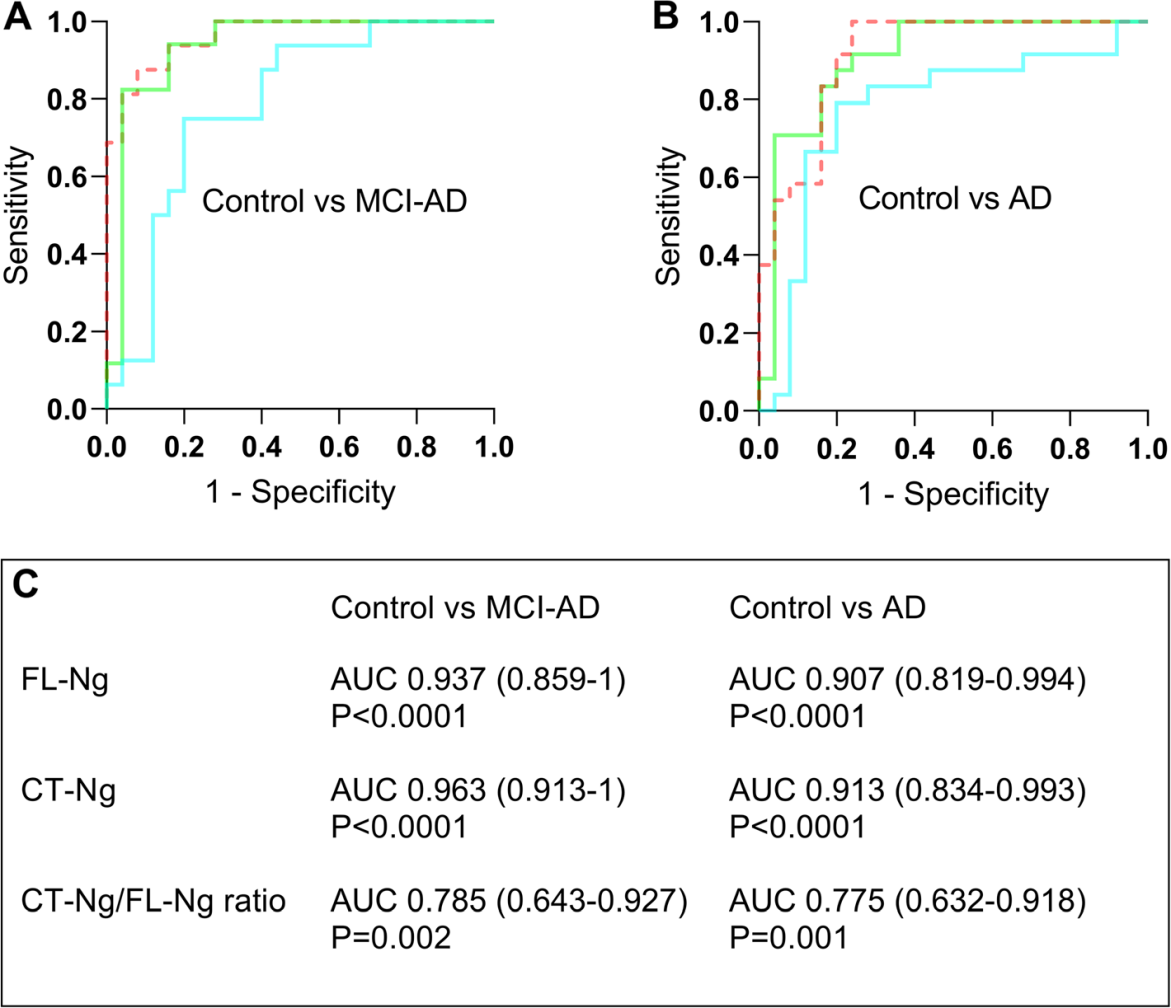
ROC curve analysis for full-length neurogranin (FL-Ng) (green), C-terminal half of Ng (CT-Ng) (orange), and the ratio of CT-Ng/FL-Ng (turquoise) in the cerebrospinal fluid (CSF) for differentiation of mild cognitive impairment due to Alzheimer’s disease (MCI-AD) from controls (**a**) and Alzheimer’s disease (AD) patients from controls (**b**). The area under the curve (95% confidence interval) is shown in the incorporated table (**c**)

FL-Ng and CT-Ng could differentiate AD from controls with AUC = 0.907 (95% CI, 0.819–0.994) and AUC = 0.913 (95% CI, 0.913–0.993), respectively (Fig. 2). Furthermore, FL-Ng (AUC = 0.937; 95% CI, 0.859–1) and CT-Ng (AUC = 0.963, 95% CI, 0.913–1) could also differentiate MCI-AD from controls (Fig. 2). The CSF ratio of CT-Ng/FL-Ng did not improve the separation either for AD (AUC = 0.775, 95% CI, 0.632–0.918) or MCI-AD (AUC = 0.785, 95% CI, 0.643–0.927) from controls (Fig. 2).

There were no statistically significant correlations between MMSE or age and the CSF levels of either FL-Ng or CT-Ng in any of the investigated groups (Table 2).

**Table 2:** Correlation between cerebrospinal fluid FL-Ng, CT-Ng, age, MMSE, and biomarker levels for the clinical study^a^

	FL-Ng	FL-Ng	FL-Ng
	**Control (*N*** **= 26)**	**MCI-AD (*N*** **= 18)**	**AD (*N*** **= 23)**
Age	rho = − 0.136, N.S.	rho = 0.430, N.S.	rho = 0.061, N.S.
MMSE	rho = − 0.001, N.S.	rho = 0.172, N.S.	rho = − 0.241, N.S.
Aβ_1–42_	rho = 0.655, *P* = 0.0003	rho = 0.351, N.S.	rho = −0.017, N.S.
T-tau	rho = 0.739, *P* = 0.00002	rho = 0.268, N.S.	rho = 0.500, *P* = 0.015
P-tau	rho = 0.627, *P* = 0.001	rho = 0.257, N.S.	rho = 0.391, N.S.
	CT-Ng	CT-Ng	CT-Ng
	**Control (*N*** **= 26)**	**MCI-AD (*N*** **= 18)**	**AD (*N*** **= 23)**
Age	rho = −0.044, N.S.	rho = 0.010, N.S.	rho = 0.043, N.S.
MMSE	rho = 0.077, N.S.	rho = 0.245, N.S.	rho = −0.132, N.S.
Aβ_1–42_	rho = 0.782, *P* = 0.0003	rho = 0.194, N.S.	rho = 0.032, N.S.
T-tau	rho = 0.787, *P* < 0.00001	rho = 0.326, N.S.	rho = 0.664, *P* = 0.0006
P-tau	rho = 0.677, *P* = 0.0002	rho = 0.621, *P* = 0.008	rho = 0.728, *P* = 0.00008

^a^Correlations presented by Spearman’s rank correlation coefficient (rho). Non-significant (N.S.) correlation coefficients (*P* > 0.05)

### The association of CSF Ng and other CSF biomarkers

The CSF levels of FL-Ng and CT-Ng significantly correlated with all core biomarkers, i.e., Aβ_1–42_ (rho = 0.655, *P* = 0.003 and rho = 0.782, *P* = 0.003), T-tau (rho = 0.739, *P* = 0.00002 and rho = 0.787, *P* < 0.00001), and P-tau (rho = 0.627, *P* = 0.001 and rho = 0.677, *P* = 0.0002) in the control group (Table 2). CT-Ng significantly correlated positively with P-tau (rho = 0.621, *P* = 0.008) in patients with MCI-AD (Table 2). FL-Ng and CT-Ng significantly correlated positively with T-tau (rho = 0.500, *P* = 0.015 and rho = 0.664, *P* = 0.0006) in AD patients, while only CT-Ng significantly correlated with P-tau (rho = 0.728, *P* = 0.00008) within this group (Table 2).

The CSF level of FL-Ng and CT-Ng significantly correlated with each other (rho = 0.815, *P* < 0.00001) and with all of the previously investigated pre-synaptic markers, i.e., SNAP-25 (rho = 0.601, *P* = 0.001 and rho = 0.736, *P* = 0.00002), synaptotagmin, 215–223 (rho = 0.642, *P* = 0.0004 and rho = 0.815, *P* < 0.00001), and synaptotagmin, 238–245 (rho = 0.640, *P* = 0.0004 and rho = 0.805, *P* < 0.00001) [6, 7] in the control group (Table 3). CT-Ng significantly correlated with synaptotagmin 215–223 (rho = 0.512, *P* = 0.04) and synaptotagmin 238–245 (rho = 0.527, *P* = 0.03) in patients with MCI-AD, while FL-Ng did not correlate with any of the pre-synaptic markers within this group (Table 3). The CSF level of CT-Ng significantly correlated with of all synaptic markers FL-Ng (rho = 0.595, *P* = 0.003), SNAP-25 (rho = 0.636, *P* = 0.001), synaptotagmin, 215–223 (rho = 0.677, *P* = 0.0004), and synaptotagmin, 238–245 (rho = 0.702, *P* = 0.0002) [6, 7] in AD patients. The CSF levels of FL-Ng significantly correlated positively with all synaptic markers CT-Ng (rho = 0.595, *P* = 0.003), synaptotagmin, 215–223 (rho = 0.577, *P* = 0.004), and synaptotagmin, 238–245 (rho = 0.585, *P* = 0.003) [6, 7], except from SNAP-25 in AD patients (Table 3).

**Table 3:** Correlation between cerebrospinal fluid FL-Ng, CT-Ng, SNAP-25, synaptotagmin, 215–223, and synaptotagmin, 238–245 levels for the clinical study^a^

	FL-Ng	FL-Ng	FL-Ng
	**Control (*N*** **= 26)**	**MCI-AD (*N*** **= 18)**	**AD (*N*** **= 23)**
CT-Ng	rho = 0.815, *P* < 0.00001	rho = 0.279, N.S.	rho = 0.595, *P* = 0.003
SNAP-25	rho = 0.601, *P* = 0.001	rho = 0.329, N.S.	rho = 0.306, N.S.
Synaptotagmin, 215–223	rho = 0.642, *P* = 0.0004	rho = 0.340, N.S.	rho = 0.577, *P* = 0.004
Synaptotagmin, 238–245	rho = 0.640, *P* = 0.0004	rho = 0.344, N.S.	rho = 0.585, *P* = 0.003
	CT-Ng	CT-Ng	CT-Ng
	**Control (*N*** **= 26)**	**MCI-AD (*N*** **= 18)**	**AD (*N*** **= 23)**
FL-Ng	rho = 0.815, *P* < 0.00001	rho = 0.279, N.S.	rho = 0.595, *P* = 0.003
SNAP-25	rho = 0.736, *P* = 0.00002	rho = 0.458, N.S.	rho = 0.636, *P* = 0.001
Synaptotagmin, 215–223	rho = 0.815, *P* < 0.00001	rho = 0.512, *P* = 0.04	rho = 0.677, *P* = 0.0004
Synaptotagmin, 238–245	rho = 0.805, *P* < 0.00001	rho = 527, *P* = 0.03	rho = 0.702, *P* = 0.0002

^a^Correlations presented by Spearman’s rank correlation coefficient (rho). Non-significant (N.S.) correlation coefficients (*P* > 0.05)

## Discussion

In the present study, we have evaluated two novel ultrasensitive Simoa assays for full-length neurogranin (FL-Ng) and C-terminal neurogranin (CT-Ng). Most of the current available quantitative Ng immunoassays [4, 9, 34, 50] contain antibodies recognizing the C-terminal half of Ng (CT-Ng), and herein we confirmed that CT-Ng is a reliable CSF biomarker to discriminate cognitive impairment (AD and MCI-AD) from controls [4, 9]. Furthermore, in a novel approach, we demonstrate that the low abundant CSF FL-Ng was also significantly elevated in both AD and MCI-AD as compared to controls. The CT-Ng and FL-Ng immunoassays had similar diagnostic performances. The ratio of CT-Ng/FL-Ng did not improve the separation either for AD from controls or MCI-AD from controls. Overall, our results agree with previously studies demonstrating that CSF Ng to be a suitable biomarker for separation of AD patients from controls, as well as reflecting the synaptic degeneration that occurs in brain early in the disease process [4, 9, 34, 35, 41, 50].

In this study, we found that the absolute CSF CT-Ng concentrations were considerably higher than the CSF FL-Ng. Since the FL-Ng assay targets aa sequences close to the N-terminal of Ng, these results are in agreement with our previously findings demonstrating that Ng is metabolized into several endogenous short C-terminal peptides that results to the markedly increased CSF levels of CT-Ng in AD patients [4]. The divergent absolute CSF FL-Ng and CT-Ng concentrations in all investigated groups may reflect that the C-terminal peptides more easily passes the neuronal cell-membranes than the longer FL-Ng and/or that those Ng peptides indeed are more abundant, especially in AD. We recently identified that Ng is cleaved in the middle of IQ motif by Calpain-1 [33], which is an enzyme interestingly known to be upregulated in AD [51, 52] and can cause the elevated CSF levels of CT-Ng in disease. Aside from a considerably lower concentration of FL-Ng that in contrast to CT-Ng contains an intact IQ domain, we also found elevated FL-Ng levels in AD compared with controls. However, the very low levels of FL-Ng, particularly in controls, might imply that CT-Ng are more prone to be liberated into CSF than FL-Ng. Discrepancies in measured levels of Ng peptides can be due to the intrinsic biochemical properties of endogenous Ng peptides of various lengths, such as differences in isoelectric points, post-translationally modifications, and disulfide linkages. For instance, Ng contains four cysteines (Cys3, Cys4, Cys9, and Cys51) that in absence of reducing agents are oxidized in vitro [53]. Since the novel Simoa assay do not contain reducing agents, the putative intracellular disulfide linkages of CSF Ng might diminish the ability of the Ng assay antibodies from binding. We have previously found several post-translational modifications on brain Ng_1–78_ that include disulfide bridges between Cys3 and Cys4 or between Cys4 and Cys9 [47]. Former mutation studies of individual Cys indicates that Cys 51 preferentially forms an intramolecular disulfide bond with Cys9 [54, 55], which perhaps make it more challenging to measure CSF FL-Ng than CT-Ng with the present non-reducing assay format. However, since reducing agents can disrupt disulfide bridges also between the polypeptide chains of assay antibodies, it was not possible to rule out that intramolecular disulfide Ng formation could influence the quantification of CSF FL-Ng and CT-Ng.

CSF CT-Ng and CSF FL-Ng levels correlated with the levels of T-tau, P-tau, and Aβ_1–42_ in the control group. While no association was found with Aβ_1–42_ in MCI-AD and AD, associations were found with tau (P-tau > T-tau) in cognitive impairment groups. Taken together, all these results support the notion that the CSF Ng is a sensitive AD biomarker (relationship to P-tau) that to some extent reflects general neurodegeneration (relationship to T-tau) [4, 34].

A further analysis in this study investigated the relationship between the CSF levels of the postsynaptic Ng and the pre-synaptic markers (SNAP-25, Synaptotagmin-1, 215–223, and Synaptotagmin-1, 238–245) [6, 7]. We found that all synaptic markers correlated with each other in the control group, while nearly all synaptic markers, except from SNAP-25 and FL-Ng, correlated with each other in the AD group. This might reflect that both the investigated pre-synaptic activity-related markers and postsynaptic Ng are associated with each other both in health and synaptic degeneration. It is merely possible to speculate on the lack of correlation of FL-Ng and SNAP-25 in AD patients. FL-Ng in contrary to CT-Ng does not correlate with P-Tau in AD, which may imply that the Ng species mirror different pathological aspects of disease. In contrast to the other synaptic proteins, SNAP-25 has both pronounced pre-synaptic functions and emerging postsynaptic roles [56, 57], and the dual release sites might affect the levels of SNAP-25. Further studies that investigate a panel of CSF synaptic markers in parallel with measure synapse density in the brain of a living patient via neuroimaging markers (e.g., synaptic vesicle glycoprotein 2A (SV2A) PET) would be valuable in order to evaluate different aspects of the CSF synaptic markers [58].

Many of the proteins associated with the AD pathogenesis are found in truncated and post-translationally modified forms in CSF [2, 4–6, 39, 59–61]. Combinations of disease-related AD CSF protein/peptide biomarkers into a ratio have shown to increase the diagnostic performance, e.g., ratio of Aβ_1–42_/Aβ_1–40_ increases the ability to distinguish patients with AD from other dementia disorders by normalization to the Aβ production (Aβ_1–40_) [40]. We have previously found that Ng peptides to total full-length ratios for nine C-terminal peptides, investigated with immuno-enrichment and mass spectrometry, were decreased in post-mortem human brain tissue from sporadic AD compared to controls [47]. Herein, applying the novel Simoa assays, we found the CSF ratio of CT-Ng/FL-Ng significantly decreased in all the examined patients’ groups compared to controls. The levels of CT-Ng and FL-Ng, respectively, separated AD patients from controls and MCI-AD from controls with high accuracy (AUC > 0.9), while the ratio of CT-Ng/FL-Ng were substantially lower (AUC < 0.8).

The major strength of our study is the establishment of two novel ultrasensitive Simoa immunoassays for the high-throughput assessment of different peptide species of Ng in CSF. The novel Simoa assays exhibit high intra-assay repeatability and inter-assay precision, not exceeding a coefficient of variation of 7% and 14%, respectively. Thus, the repeatability was within acceptable ranges [49] to use in larger research cohort and therapeutic trials.

### Limitations

There are some limitations of our study. Firstly, previous studies have shown that CSF Ng may serve as a biomarker of cognition [4, 34, 35, 62]. In this study, however, no correlation between FL-Ng or CT-Ng with cognitive decline, indexed by MMSE, in any of the examined groups was observed. The lack of evidence for correlation between cognition and Ng can be due to the low number of participants within each diagnostic category. The cross-sectional design may also prevent the confirmation of CSF Ng as prognostic marker of cognitive decline. Furthermore, the MMSE has limitations as sensitive measures for specific cognitive domains. A cognitive test that directly evaluates executive function (e.g., Trail Making Test B, Stroop Test, Verbal Fluency Test) may have yield a greater correlation. Secondly, antibodies of our novel assays are not end-specific; thus, we could not rule out that probable longer Ng fragments beyond the antibody-defined epitopes can be quantified. Lastly, Ng peptides of various lengths can have different post-translational modifications, disulfide linkages, and isoelectric points that might affect the measured levels.

## Conclusions

In summary, we have applied the ultrasensitive Simoa technology to develop two novel immunoassays for assessment of the CSF levels of FL-Ng and CT-Ng. Evaluation of these assays showed significantly increased CSF levels of FL-Ng and CT-Ng in AD compared to controls, but also in MCI-AD patients. In contrast, the ratio of CT-Ng/FL-Ng was decreased in AD and in MCI-AD but was found to be less sensitive to detect MCI-AD and AD than single biomarkers. Altogether, these results add further evidence to the notion of CSF Ng as a reliable biomarker for AD and early on in the pathogenesis. The novel and robust Simoa assays will be useful tools in future investigations of synaptic changes in CSF in intervention therapeutic studies with novel disease-modifying drug candidates.

## Data Availability

The datasets used and/or analyzed during the present study are available from the corresponding author on reasonable request.

## Abbreviations

aa: Amino acids
Aβ: Amyloid-β
AD: Alzheimer’s disease
AUC: Area under the curve
CI: Confidence interval
CNS: Central nervous system
CSF: Cerebrospinal fluid
CT-Ng: C-terminal neurogranin
ELISA: Enzyme-linked immunosorbent assay
FL-Ng: Full-length neurogranin
LOD: Limit of detection
LTP: Long-term potentiation
MCI: Mild cognitive impairment
MMSE: Mini-Mental State Examination
NFT: Neurofibrillary tangles
Ng: Neurogranin
PBS: Phosphate-buffered saline
PET: Positron emission tomography
P-tau: Tau phosphorylated at threonine 181
QC: Quality control
ROC: Receiver operating characteristic
Simoa: Single molecule array
SNAP-25: Synaptosomal-associated protein 25
SV2A: Synaptic vesicle glycoprotein 2A
T-Tau: Total tau
